# Dexamethasone Attenuates the Expression of MMP-13 in Chondrocytes through MKP-1

**DOI:** 10.3390/ijms23073880

**Published:** 2022-03-31

**Authors:** Tiina Lehtola, Elina Nummenmaa, Lauri Tuure, Mari Hämäläinen, Riina M. Nieminen, Teemu Moilanen, Antti Pemmari, Eeva Moilanen

**Affiliations:** 1 The Immunopharmacology Research Group, Faculty of Medicine and Health Technology, Tampere University and Tampere University Hospital, 33014 Tampere, Finland; tiina.lehtola@tuni.fi (T.L.); elina.nummenmaa@tuni.fi (E.N.); lauri.tuure@tuni.fi (L.T.); mari.hamalainen@tuni.fi (M.H.); riinamaria.nieminen@gmail.com (R.M.N.); teemu.moilanen@coxa.fi (T.M.); antti.pemmari@tuni.fi (A.P.); 2 Coxa Hospital for Joint Replacement, 33520 Tampere, Finland

**Keywords:** MKP-1, MMP-13, osteoarthritis, chondrocyte, inflammation, dexamethasone

## Abstract

Mitogen-activated protein kinase phosphatase-1 (MKP-1) is upregulated in inflammation and reduces the activity of proinflammatory mitogen-activated protein kinases (MAP kinases) by dephosphorylation. MAP kinases are intracellular signaling pathways that mediate the cellular effects of proinflammatory cytokines. In the present study, we investigated the effects of the glucocorticoid dexamethasone on the expression of catabolic enzymes in chondrocytes and tested the hypothesis that these effects are mediated through MKP-1. Dexamethasone was found to significantly attenuate the expression of matrix metalloproteinase (MMP)-13 in human OA chondrocytes as well as in chondrocytes from MKP-1 WT mice, but not in chondrocytes from MKP-1 KO mice. Dexamethasone also increased the expression of MKP-1 in murine and human OA chondrocytes. Furthermore, p38 MAP kinase inhibitors significantly attenuated MMP-13 expression in human OA chondrocytes, while JNK MAP kinase inhibitors had no effect. The results indicate that the effect of dexamethasone on MMP-13 expression in chondrocytes was mediated by an MKP-1 and p38 MAP kinase-dependent manner. These findings, together with previous results, support the concept of MKP-1 as a protective factor in articular chondrocytes in inflammatory conditions and as a potential drug target to treat OA.

## 1. Introduction

Osteoarthritis (OA) is the most common joint disease, and a leading cause of disability worldwide [1]. The prevalence of OA is constantly rising due to increasing age and obesity [2]. OA is a slowly progressing inflammatory joint disease that involves cartilage degradation, synovial inflammation, joint deformity and pain [1,2,3,4,5]. OA affects the entire joint structure, but the most severe changes are seen in cartilage, which is affected by degradative factors produced in all tissues of the joint [6]. Chondrocytes, the only cell type present in articular cartilage, are considered to be essential in the pathogenesis of OA [1]. Increased amounts of inflammatory mediators and cartilage matrix-degrading enzymes are produced by chondrocytes during the progression of OA, leading to an imbalance between the production of anabolic vs. catabolic and inflammatory factors within the OA joint [4]. Current therapies for OA are limited, and there is a major unmet need for drugs and other treatment modalities which could enable the prevention, retardation or repair of cartilage degradation or injury [5,7,8,9].

The proinflammatory cytokines interleukin-1β (IL-1β), tumor necrosis factor α (TNF-α) and interleukin-6 (IL-6) are considered the most important inflammatory mediators driving the pathogenesis of OA and associated cartilage degradation [1]. These inflammatory mediators stimulate the production of matrix-degrading enzymes including various ADAMTSs (a disintegrin and metalloproteinase with thrombospondin motifs) and MMPs (matrix metalloproteinases) [4]. MMP-13 is regarded as one of the most important MMP enzymes in OA [6] and is found at elevated levels in the joints of OA patients [10,11,12,13]. MMP-13 efficiently degrades interstitial type II collagen, which is the main collagen component in articular cartilage [6].

IL-1β activates mitogen-activated protein kinase (MAP kinase) signaling [1]. MAP kinases are a family of intracellular signaling proteins that regulate many physiological cellular processes [14]. Several MAP kinases have been identified, and p38 and c-Jun N-terminal kinase (JNK) MAP kinases are known to have an important role in the immune response and regulation of inflammation [14]. Activation of MAP kinases is tightly regulated by phosphorylation of threonine and tyrosine residues in the activation domain of the kinase [15]. Activated MAP kinases enhance the expression of their target genes through phosphorylation of transcription factors and other regulatory proteins [16]. Mitogen-activated protein kinase phosphatase 1 (MKP-1) is one of the key enzymes that regulate the activity of p38 and JNK MAP kinases by dephosphorylation [14,17]. By inhibiting these MAP kinases, MKP-1 has been proposed to suppress the expression of inflammatory genes and to attenuate acute and chronic inflammatory responses [14]. Several anti-inflammatory drugs (e.g., glucocorticoids, β2-receptor agonists, phosphodiesterase 4 (PDE4) inhibitors and the antirheumatic drug aurothiomalate) have been reported to enhance MKP-1 expression, and at least part of the anti-inflammatory effects of those drugs may be mediated by MKP-1 [15,18,19,20].

Glucocorticoids are effective anti-inflammatory drugs widely used to treat arthritis and various other inflammatory diseases [21]. Glucocorticoids generally exert their effects by binding to the glucocorticoid receptor (GR), which then forms a homodimer that is transported to the nucleus, where it binds to the glucocorticoid-responsive element (GRE) to upregulate the transcription of its target genes [22]. Interestingly, GRE has been identified in the promoter region of the MKP-1 gene [15].

We have recently shown that the glucocorticoid dexamethasone has a widespread effect on the gene expression of primary human OA chondrocytes [23]. We hypothesized that the effects of dexamethasone on gene expression in chondrocytes could be at least partly mediated through MKP-1. The aim of the present study was to test this hypothesis focusing specifically on MMP-13, which has been shown to be one of the key cartilage matrix-degrading enzymes in OA.

## 2. Results

### 2.1. Dexamethasone Attenuates the Expression of Catabolic Enzymes in Chondrocytes from MKP-1 WT Mice, and This Effect Is Diminished in MKP-1 KO Mice

Genome-wide expression analysis (RNA-Seq) was carried out to investigate the effects of dexamethasone in chondrocytes from MKP-1 KO and corresponding WT mice. Among the detected genes, we were interested in those associated with cartilage degradation in OA. Dexamethasone was found to attenuate the expression of several *Mmp* and *Adamts* genes in chondrocytes obtained from WT mice. Interestingly, the effects of dexamethasone on these genes were largely reduced in chondrocytes from MKP-1 KO mice (Table 1). Notably, the expression of OA-related cartilage matrix-degrading enzyme MMP-13 was significantly attenuated in chondrocytes from MKP-1 WT mice, but no effect was seen in chondrocytes from MKP-1 KO mice.

### 2.2. Dexamethasone Attenuates MMP-13 Expression in IL-1β-Stimulated Chondrocytes from WT Mice, but Not in Chondrocytes from MKP-1 KO Mice

**Figure 1:**
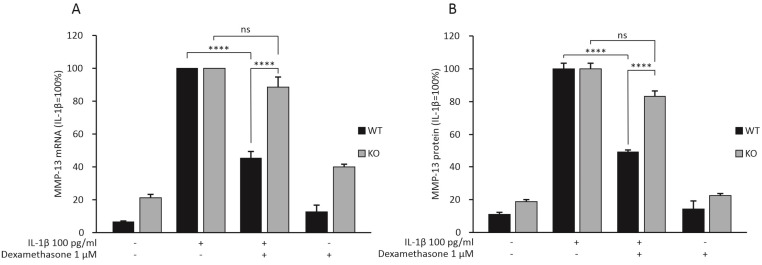
Dexamethasone attenuated the expression of MMP-13 mRNA (**A**) and protein (**B**) in primary chondrocytes from MKP-1 WT mice, but not in chondrocytes from MKP-1 KO mice. Chondrocytes were treated with IL-1β (100 pg/mL) alone or together with dexamethasone (1 μM) for 24 h. The mRNA levels of MMP-13 were measured with quantitative RT-PCR and normalized against GAPDH mRNA levels. The MMP-13 protein levels were determined with ELISA. MMP-13 expression levels in IL-1β-treated samples of each genotype were set at 100%, and all other values are expressed in relation to those values. The results are expressed as mean + SEM, *n* = 8. Two-way ANOVA followed by Tukey’s post-test was performed; **** *p* < 0.0001, ns: not significant.

MMP-13 has been shown to be one of the most important enzymes in OA-associated cartilage degradation [6]. In the RNA-Seq analysis, dexamethasone attenuated *Mmp-13* expression in chondrocytes from WT mice, but not from MKP-1 KO mice. Therefore, we wanted to confirm this finding in another set of chondrocytes obtained from MKP-1 KO and corresponding WT mice. In accordance with the RNA-Seq results, dexamethasone reduced IL-1β-stimulated mRNA (Figure 1A) and protein (Figure 1B) expression of MMP-13 in chondrocytes from WT mice but not in chondrocytes from MKP-1 KO mice. In addition, *Mmp-13* expression was about two-fold higher in MKP-1 KO than in WT chondrocytes. These results indicate that MKP-1 was involved in regulating MMP-13 expression in chondrocytes.

### 2.3. Dexamethasone Increases the Expression of MKP-1 in Chondrocytes

**Figure 2:**
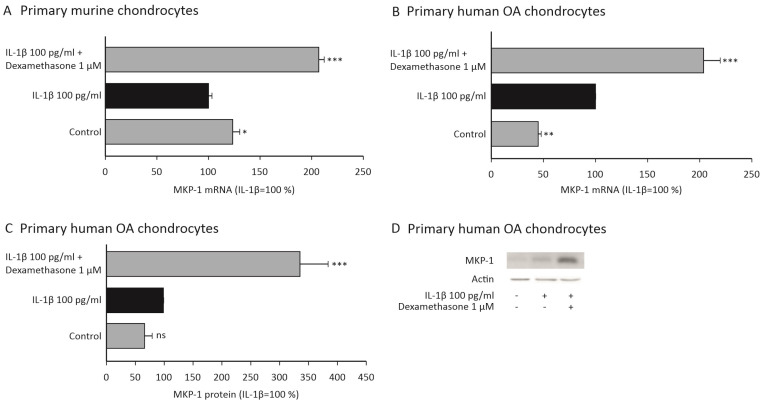
Dexamethasone increased the expression of MKP-1 in IL-1β-stimulated primary murine (**A**) and human OA (**B**–**D**) chondrocytes. (**A**–**C**) Primary murine and human OA chondrocytes were treated with IL-1β (100 pg/mL) alone or together with dexamethasone (1 μM) for 90 min. In (**A**,**B**), the mRNA levels of MKP-1 were measured with qRT-PCR and normalized against GAPDH mRNA levels. In (**C**), protein levels of MKP-1 and actin were determined by Western blotting. MKP-1 protein expression levels were normalized against actin levels. In (**A**–**C**), normalized MKP-1 expression levels in IL-1β-stimulated samples were set as 100%, and all other values are expressed in relation to those values. The results are expressed as mean + SEM, *n* = 4–6. Analysis of variance followed by Bonferroni post-test was performed; * *p* < 0.05, ** *p* < 0.01, *** *p* < 0.001. In (**D**), one representative Western blot result is shown.

Dexamethasone was found to downregulate the expression of MMP-13 in chondrocytes from wild-type mice but not in cells from MKP-1-deficient mice. We wanted to further investigate whether dexamethasone upregulates MKP-1 expression, to support the hypothesis that these effects are mediated through MKP-1. We found that dexamethasone did indeed increase MKP-1 expression in IL-1β-stimulated murine (Figure 2A) and human OA (Figure 2A–D) chondrocytes.

### 2.4. Dexamethasone and p38 MAP Kinase Inhibitors Attenuate the Expression of MMP-13 in Primary Human OA Chondrocytes

**Figure 3:**
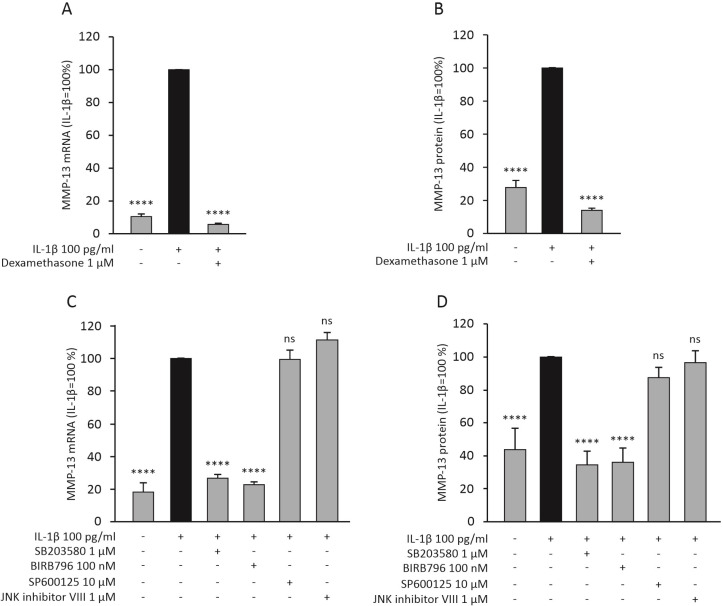
Dexamethasone (**A**,**B**) and p38 MAP kinase inhibitors (**C**,**D**) reduced the expression of MMP-13 mRNA and protein in human OA chondrocytes. In A and B, the chondrocytes were treated with IL-1β (100 pg/mL) alone and together with dexamethasone (1 μM) for 24 h. In (**C**,**D**), the chondrocytes were treated with IL-1β (100 pg/mL) alone and together with the selective p38 MAP kinase inhibitors SB203580 (1 µM) or BIRB796 (100 nM), or with the selective JNK inhibitors SP600125 (10 µM) or JNK inhibitor VIII (1 µM) for 24 h. The mRNA levels of MMP-13 were measured with quantitative RT-PCR and normalized against GAPDH mRNA levels. MMP-13 protein levels were determined with ELISA. MMP-13 expression levels in IL-1β-treated samples were set at 100%, and all other values are presented in relation to those values. The results are given as mean + SEM, *n* = 8 (**A**,**B**) or *n* = 5 (**C**,**D**). Samples were obtained from eight (**A**,**B**) or five (**C**,**D**) patients with OA, and the experiments were carried out in duplicate. Repeated measures ANOVA followed by Bonferroni post-test was performed; **** *p* < 0.0001, ns = not significant.

Dexamethasone significantly attenuated the expression of MMP-13 also in human OA chondrocytes at the mRNA (Figure 3A) and protein (Figure 3B) level. Since dexamethasone was found to attenuate MMP-13 expression through MKP-1, and MKP-1 is known to dephosphorylate p38 and JNK MAP kinases, we further wanted to investigate if these MAP kinases have an effect on MMP-13 expression. p38 MAP kinase inhibitors SB203580 and BIRB796 were found to attenuate the expression of MMP-13 mRNA (Figure 3C) and protein (Figure 3D) in human OA chondrocytes. Interestingly, JNK inhibitors SP600125 and JNK inhibitor VIII did not have an effect on MMP-13 expression (Figure 3C,D). Together these findings suggest that p38 MAP kinase could be involved in the regulation of MMP-13 expression in these cells, while JNK kinase may not have a significant role.

## 3. Discussion

In the present study, we discovered that the glucocorticoid dexamethasone attenuated the expression of MMP-13 in chondrocytes through MKP-1. This effect was potentially mediated through inactivation of p38 MAP kinase. According to RNA-seq analysis, dexamethasone also altered the expression of several other genes related to cartilage degradation in an MKP-1-dependent manner. Moreover, dexamethasone increased MKP-1 expression in chondrocytes.

MMP-13 (also known as collagenase 3) is one of the major cartilage matrix-degrading enzymes in OA. MMP-13 efficiently degrades type II collagen, which is the most abundant collagen type in articular cartilage [6]. MMP-13 is produced by human and murine articular chondrocytes and is found at elevated levels in the cartilage of OA patients [24,25,26]. The importance of MMP-13 in OA progression has also been shown in experimental models of OA. Using the surgical destabilization of the medial meniscus (DMM) model as well as combinations of ligament transection and meniscectomy models of OA, MMP-13 has been shown to be overexpressed in osteoarthritic cartilage, and structural damage in cartilage has been shown to be significantly worse in WT mice compared to that in MMP-13 KO mice [27,28]. Considering the catabolic effects of MMP-13 in OA cartilage, it appears to be a promising drug target. Selective MMP-13 inhibitors are currently under development for the treatment of OA [6].

Glucocorticoids are broad-spectrum anti-inflammatory drugs widely used in the treatment of inflammatory conditions [21]. In osteoarthritis, they are administered as intra-articular injections during exacerbation phases [29]. They exert their effects by suppressing the expression of proinflammatory genes and by enhancing the expression of anti-inflammatory factors, such as MKP-1 [21]. We have previously shown that the glucocorticoid dexamethasone has widespread effects on gene expression of human OA chondrocytes [23]. Glucocorticoids are known to enhance the expression of MKP-1 in chondrocytes, macrophages [30,31], airway epithelial and smooth muscle cells [32,33] and endothelial cells [15]. In accordance with these previous findings, we showed in the present study that dexamethasone significantly enhanced the expression of MKP-1 in both murine and human chondrocytes. The promoter region of MKP-1 gene contains binding cites for several transcription factors, including activator protein 1 (AP-1), NF-κB, cAMP response element-binding protein (CREB) in addition to the glucocorticoid-responsive element (GRE) [15]. Accordingly, several other anti-inflammatory drugs, such as β2-receptor agonists, phosphodiesterase 4 (PDE4) inhibitors and aurothiomalate, have also been shown to enhance MKP-1 expression [18,19,20,34].

In the present study, the glucocorticoid dexamethasone significantly attenuated the expression of MMP-13 in chondrocytes from WT mice, but not in chondrocytes from MKP-1 KO mice. This strongly suggests that the effect of dexamethasone is mediated by MKP-1. We also showed that dexamethasone enhanced the expression of MKP-1 in primary chondrocytes. MKP-1 (also referred to as dual specificity phosphatase 1 (DUSP1)) has a significant role in the regulation of innate and adaptive immune responses, including inflammatory reactions [15]. Studies using MKP-1 KO mice have shown that MKP-1 suppresses the expression of inflammatory genes (such as TNF, IL-6, COX-2) and attenuates acute and chronic inflammatory responses [35,36,37,38,39,40,41,42,43]. MKP-1 dephosphorylates the threonine and tyrosine residues in the activation domain of p38 and JNK MAP kinases, thus decreasing the activity of the kinase [11], and we have recently shown that also in human OA chondrocytes [42]. Therefore, we aimed to investigate whether MMP-13 expression is mediated through one or both of these MAP kinases. Two p38 MAP kinase inhibitors, SB203580 and BIRB796, significantly reduced MMP-13 expression in human OA chondrocytes. In contrast, the JNK kinase inhibitors SP600125 and JNK inhibitor VIII did not have an effect. p38 MAP kinase has also previously been shown to mediate enhanced MMP-13 expression in chondrocytes [44,45,46]. These findings suggest that regulation of MMP-13 expression in chondrocytes by MKP-1 is mediated through p38, but not JNK MAP kinase.

**Figure 4:**
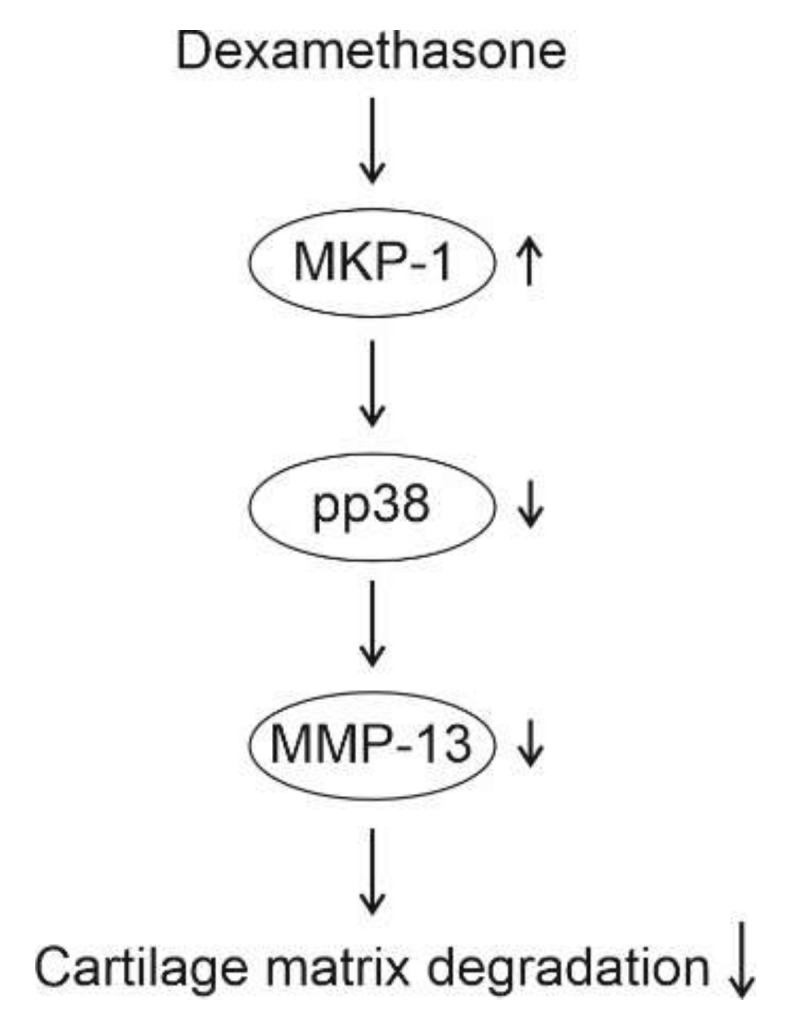
Schematic presentation of the mechanism of action of dexamethasone in chondrocytes according to the present study. Dexamethasone enhances the expression of MKP-1, leading to dephosphorylation (i.e., inactivation) of p38 MAP kinase. Decreased activity of p38 MAP kinase attenuates MMP-13 expression, resulting in decreased cartilage matrix degradation.

The present study shows that the glucocorticoid dexamethasone significantly inhibited MMP-13 expression in primary murine and human OA chondrocytes, and that effect was abolished in chondrocytes from MKP-1-deficient mice, strongly suggesting that the observed effect was mediated through MKP-1. The results further indicate that this effect may be mediated through inhibition of p38 MAP kinase. Dexamethasone was also shown to increase the expression of MKP-1 in chondrocytes under inflammatory conditions. A schematic presentation of the mechanism of the inhibitory effect of dexamethasone on MMP-13 expression based on the present results is shown in Figure 4. These findings suggest that the favorable effects of glucocorticoids on chondrocyte gene expression are partly mediated through MKP-1. The results of this study, together with previous findings on the MKP-1-mediated anti-inflammatory effects, support MKP-1 as a potential novel drug target in the treatment of OA.

## 4. Materials and Methods

### 4.1. Animals

MKP-1 knockout (KO) (-/-) C57BL/6 mice originally generated in the laboratory of R. Bravo at Bristol-Myers Squibb Pharmaceutical Research Institute (Princeton, NJ, USA) were used [47]. The animals were housed and handled according to the legislation for the protection of animals used for scientific purposes (Directive 2010/63/EU, 22 September 2010).

### 4.2. Mouse Chondrocyte Isolation and Culture

After the mice were euthanized, full-thickness articular cartilage from the femoral heads was removed aseptically. For enzymatic isolation, the cartilage pieces were placed into a petri dish containing culture medium (DMEM (Sigma-Aldrich, St. Louis, MO, USA) supplemented with penicillin (100 U/mL), streptomycin (100 µg/mL) and amphotericin B (250 ng/mL), all from Gibco/Life Technologies, Carlsbad, CA, USA) containing Collagenase D enzyme (3 mg/mL, Sigma-Aldrich) and incubated o/n at 37 °C in 5% CO_2_. After incubation, the contents of the petri dish were mixed rigorously to detach cells from residual cartilage pieces, and the mixture was filtered through a 70 µM cell strainer to obtain a single cell suspension. The cells were washed once with culture medium, after which they were pelleted, resuspended in culture medium, and the cell number was counted [48].

Isolated chondrocytes were seeded on 24-well plates (2.0 × 10^5^ cells/mL) in Dulbecco’s Modified Eagle’s Medium (DMEM, Sigma-Aldrich) supplemented with penicillin (100 U/mL), streptomycin (100 µg/mL) and amphotericin B (250 ng/mL), all from Gibco/Life Technologies, containing 10% fetal bovine serum (Lonza, Verviers, Belgium), and cultured for seven days before conducting the experiments. In the experiments, the cells were treated with IL-1β (R&D Systems Europe, Abingdon, UK) with or without dexamethasone (Orion Corporation, Espoo, Finland), and the expression of MMP-13 and MKP-1 were determined at indicated time points.

### 4.3. Human OA Chondrocyte Isolation and Culture

The study was approved by the Ethics Committee of Tampere University Hospital, Finland and carried out in accordance with the declaration of Helsinki. Written informed consent was obtained from the patients.

Leftover pieces of cartilage from knee replacement surgery from OA patients were processed, and chondrocytes were isolated [49]. Articular cartilage was removed aseptically from subchondral bone, cut into full-thickness pieces approximately 4 mm in diameter, and washed with PBS. The chondrocytes were enzymatically isolated by incubating the cartilage pieces in culture medium (DMEM (Sigma-Aldrich) supplemented with penicillin [100 U/mL], streptomycin [100 µg/mL] and amphotericin B [250 ng/mL], all from Gibco/Life Technologies) containing Liberase^TM^ enzyme blend (0.25 mg/mL, Roche, Mannheim, Germany) for 16 h at 37 °C in a shaker. After incubation, the cell suspension was filtered through a 70 µM cell strainer to obtain a single cell suspension and washed once with culture medium. Thereafter the cells were pelleted, resuspended in culture medium and the cell number was counted. Chondrocytes were seeded on 24-well plates (2.0 × 10^5^ cells/mL) in DMEM (Sigma-Aldrich) supplemented with penicillin (100 U/mL), streptomycin (100 µg/mL) and amphotericin B (250 ng/mL), all from Gibco/Life Technologies, containing 10% fetal bovine serum (Lonza), and cultured for 24 h before conducting the experiments. In the experiments, the cells were treated with IL-1β (R&D Systems Europe, Abingdon, UK), dexamethasone (Orion Corporation), SB203580 (p38 inhibitor, Sigma-Aldrich), BIRB796 (p38 inhibitor, Axon MedChem, Groningen, The Netherlands), SP600125 (JNK inhibitor, Sigma-Aldrich) or JNK inhibitor VIII (JNK inhibitor, Calbiochem, Darmstadt, Germany) as indicated in the results section.

### 4.4. RNA Isolation and Sample Preparation

At the indicated time points, cell culture medium was removed, and total RNA of the chondrocytes was extracted with a Qiagen RNeasy Kit (Qiagen, Hilden, Germany) and treated with RNAse free DNAse I (Qiagen). RNA concentration and integrity were determined with a 2100 Bioanalyzer (Agilent Technologies, Santa Clara, CA, USA).

### 4.5. Next-Generation Sequencing and Data Analysis

Samples for RNA sequencing were prepared from eight pools of MKP-1 KO murine chondrocytes and from eight corresponding WT ones.

The samples were sequenced at Biomedicum Functional Genomics Unit (University of Helsinki, Finland) using the llumina NextSeq 500 System with a sequencing depth of 20 M paired-end reads, 75 bp in length. Read quality was evaluated using FastQC [50]. The reads were trimmed with Trimmomatic and aligned to reference mouse genome (GRCm38.p6) with Spliced Transcripts Alignment to a reference (STAR) [51]. The featureCounts [52] program was used to prepare count matrices. Differential gene expression was assessed with DESeq2 [53].

### 4.6. Quantitative Reverse Transcription Polymerase Chain Reaction (qRT-PCR)

Total RNA was extracted as described above and reverse transcribed to cDNA using a Maxima First Strand cDNA synthesis kit (Thermo Fisher Scientific, Waltham, MA, USA) or with Taqman Reverse Transcription reagents and random hexamers (Applied Biosystems, Foster City, CA, USA). Quantitative PCR was performed using TaqMan Universal PCR Master Mix and the ABI 7500 Real-Time PCR system (Applied Biosystems, Foster City, CA, USA). The primers and probes were purchased from Metabion (Martinsried, Germany). Their sequences (Table 2) and concentrations were optimized according to the manufacturer’s instructions in TaqMan Universal PCR Master Mix Protocol part number 4,304,449 revision C (Applied Biosystems). PCR reaction: incubation at 50 °C for two minutes, incubation at 95 °C for ten minutes and thereafter forty cycles of denaturation at 95 °C for 15 s and annealing and extension at 60 °C for one minute. The relative mRNA levels were quantified using the standard curve method, and expression levels were normalized against glyceraldehyde 3-phosphate dehydrogenase (GAPDH) mRNA levels.

### 4.7. Enzyme-Linked Immunosorbent Assay

Concentrations of MMP-13 in medium samples from human OA chondrocytes and murine MKP-1 KO and corresponding WT chondrocytes were determined by enzyme-linked immunosorbent assay (ELISA) with commercial reagents (R&D Systems Europe Ltd., Abingdon, UK).

### 4.8. Western Blot

The preparation of the cell lysates and the Western blot analysis were conducted as previously described [54]. Primary antibodies used were actin (sc-1616, Santa Cruz Biotechnology, CA, USA) and MKP-1 (SAB2500331, Sigma-Aldrich). The secondary antibody used was polyclonal goat anti-rabbit (sc-2004, Santa Cruz Biotechnology).

### 4.9. Statistics

GraphPad Prism Version 9 (GraphPad Software) was used for data analysis. Data is presented as mean + standard error of the mean (SEM). Ordinary, repeated measures and two-way ANOVA (analysis of variance), followed by Bonferroni or Tukey’s post-tests, were used in the statistical analysis where appropriate. Differences were considered significant at * *p* < 0.05, ** *p* < 0.01, *** *p* < 0.001 and **** *p* < 0.0001.

Among the genes detected in the RNA-seq analysis, we selected for Table 1 MMP and ADAMTS genes with the following criteria: mean expression levels of 20 or higher in DESeq2-normalized counts in the IL-1β-treated chondrocytes from WT mice, at least –2-fold change (FC) and FDR-corrected *p*-value of <0.05 between IL-1β- and IL-1β + dexamethasone-treated chondrocytes from WT mice. The fold change comparisons between the genotypes were calculated from log2FC and lfcSE (standard error of log2FC) values obtained from DESeq2. The statistical analysis was performed with *t*-test and Bonferroni correction for multiple analysis.

## Figures and Tables

**Table 1:** The effects of dexamethasone on the expression of selected genes related to cartilage degradation in MKP-1 knockout and corresponding wild-type mice.

Gene	Mean WT (IL-1β)	Mean WT (IL-1β + dexa)	WT FC	WT FDR *p*	Mean KO (IL-1β)	Mean KO (IL-1β + dexa)	KO FC	KO FDR *p*	*p* between the Effect of Dexamethasone in WT vs. KO Cells
**MMP-3**	173,754.8	35,166.6	–25.8	<0.0001	451,987.4	81,385.2	–5.5	<0.0001	<0.0001
**MMP-9**	126.2	28.1	–6.6	<0.0001	164.8	22.0	–6.1	<0.0001	0.72
**MMP-10**	84.7	19.1	–11.5	<0.0001	224.3	45.8	–4.7	<0.0001	0.0069
**MMP-12**	1242.1	446.7	–14.6	<0.0001	5572.8	516.9	–11.0	<0.0001	0.033
**MMP-13**	67,643.1	38,510.2	–2.3	<0.0001	62,517.4	59,714.4	–1.0	0.51	<0.0001
**ADAMTS5**	4783.2	2340.8	–3.1	<0.0001	8471.5	7194.1	–1.2	<0.0001	<0.0001
**ADAM22**	93.7	55.0	–2.0	<0.0001	56.5	26.8	–2.0	<0.0001	0.94

RNA-Seq analysis was performed on chondrocytes obtained from MKP-1 KO and corresponding WT mice. The cells were treated with IL-1β alone and together with dexamethasone. Among the genes detected in the RNA-seq analysis, we selected all the MMP and ADAMTS genes with the following criteria: mean expression levels of 20 or higher in DESeq2-normalized counts in the IL-1β-treated chondrocytes from WT mice, at least –2-fold change (FC) and FDR-corrected *p*-value of <0.05 between IL-1β- and IL-1β + dexamethasone-treated chondrocytes from WT mice. The fold change comparisons between the genotypes were calculated from log2FC and lfcSE (standard error of log2FC) values obtained from DESeq2. The statistical analysis was performed with *t*-test and Bonferroni correction for multiple analysis. FDR = false discovery rate, dexa = dexamethasone.

**Table 2:** Primers and probes used in qRT-PCR.

Primer/Probe		Sequence
mGAPDH	forward	5′-GCATGGCCTTCCGTGTTC-3′
	reverse	5′-GATGTCATCATACTTGGCAGGTTT-3′
	probe	5′-TCGTGGATCTGACGTGCCGCC-3′
mMMP-13	forward	5′-TTGTGTTTGCAGAGCACTACTTGA-3′
	reverse	5′-AACTGTGGAGGTCACTGTAGACTTCTT-3′
	probe	5′-CATCCTGCGACTCTTGCGGGAATC-3′
mMKP-1	forward	5′-CTCCTGGTTCAACGAGGCTATT-3′
	reverse	5′-TGCCGGCCTGGCAAT-3′
	probe	5′-CCATCAAGGATGCTGGAGGGAGAGTGTT-3′
hGAPDH	forward	5′-AAGGTCGGAGTCAACGGATTT-3′
	reverse	5′-GCAACAATATCCACTTTACCAGAGTTAA-3′
	probe	5′-CGCCTGGTCACCAGGGCTGC-3′
hMMP-13	forward	5′-TGATCTCTTTTGGAATTAAGGAGCAT-3′
	reverse	5′-GGAACTACTTGTCCAGGTTTCATCAT-3′
	probe	5′-CCCTCTGGCCTGCTGGCTCATG-3′
hMKP-1	forward	5′-ACGAGGCCATTGACTTCATAGAC-3′
	reverse	5′-TCGATTAGTCCTCATAAGGTAAGCAA-3′
	probe	5′-CCACTGCCAGGCAGGCATTTCC-3′

## Data Availability

All relevant data is included in the manuscript.

## Abbreviations

ANOVA	analysis of variance
ELISA	enzyme-linked immunosorbent assay
FC	fold change
FDR	false discovery rate
IL	interleukin
KO	knockout
MAP kinase	mitogen-activated protein kinase
MKP-1	mitogen-activated protein kinase phosphatase-1
MMP	matrix metalloproteinase
OA	osteoarthritis
qRT-PCR	quantitative reverse transcription polymerase chain reaction
RNA-Seq	RNA sequencing
TNF	tumor necrosis factor
WT	wild-type
